# Head-Neck Taper Corrosion in Hip Arthroplasty

**DOI:** 10.1155/2015/758123

**Published:** 2015-04-14

**Authors:** S. Hussenbocus, D. Kosuge, L. B. Solomon, D. W. Howie, R. H. Oskouei

**Affiliations:** ^1^Department of Orthopaedics and Trauma, Royal Adelaide Hospital, North Terrace, Adelaide, SA 5000, Australia; ^2^Department of Orthopaedics and Trauma, Royal Adelaide Hospital and Centre for Orthopaedic and Trauma Research, The University of Adelaide, North Terrace, Adelaide, SA 5000, Australia; ^3^School of Computer Science, Engineering and Mathematics, Flinders University, Clovelly Park, SA 5042, Australia

## Abstract

Modularity at the head-neck junction of the femoral component in THA became popular as a design feature with advantages of decreasing implant inventory and allowing adjustment of leg length, offset, and soft tissue balancing through different head options. The introduction of a new modular interface to femoral stems that were previously monoblock, or nonmodular, comes with the potential for corrosion at the taper junction through mechanically assisted crevice corrosion. The incidence of revision hip arthroplasty is on the rise and along with improved wear properties of polyethylene and ceramic, use of larger femoral head sizes is becoming increasingly popular. Taper corrosion appears to be related to all of its geometric parameters, material combinations, and femoral head size. This review article discusses the pathogenesis, risk factors, clinical assessment, and management of taper corrosion at the head-neck junction.

## 1. Introduction

Modularity at the head-neck junction of stems in total hip arthroplasty (THA) became popular in the 1980s [1]. This design feature decreases implant inventory and simplifies subsequent revision by offering the option of retaining the stem and performing a head exchange. Head modularity allows adjustments of leg length and offset [1–3] and also allows the use of ceramic as a bearing option. If the stem is to be retained at revision, exposure may be improved by head removal, which also offers the opportunity to apply a new head prior to closure. This may be beneficial in terms of subsequent bearing surface wear. Despite its potential benefits, an increase in the use of modular interfaces can lead to an increase in fretting and crevice corrosion at the taper junction [3–6].

The confirmation of corrosion at the femoral head-neck junction is made at revision surgery. The products of taper corrosion may contribute to third-body wear of the articulation. Although taper corrosion is relatively rare in hips with metal-on-polyethylene articulations, the corrosion products can lead to adverse local tissue reactions (ALTRs) of the same type as that occurring with large diameter metal-on-metal (MoM) bearings [1]. Head-neck taper corrosion is well-described in large MoM bearing surfaces [7–9] but its occurrence with conventional metal or ceramic on polyethylene bearings is still relatively scarce. Due to improved wear properties of highly cross-linked polyethylene (HXLPE) [10–12] and ceramic [13], surgeons are more accepting of thinner polyethylene liners, allowing the use of larger femoral heads to reduce the risk of dislocation [14, 15]. The number of revision THAs being performed is increasing and the higher dislocation rates in revision surgery also favour the use of larger femoral heads [14–17]. Factors affecting taper corrosion include taper design, femoral head material and size, use of dissimilar metals at the head-neck junction, lateral offset, varus femoral stem position, and time since implantation [2, 5, 6, 18–21]. This paper reviews the current literature on taper corrosion at the head-neck junction and its clinical implications. In this paper, the term head-neck junction refers to the taper between the bore of the femoral head and the trunnion of the femoral stem.

## 2. Definitions

**Figure 1:**
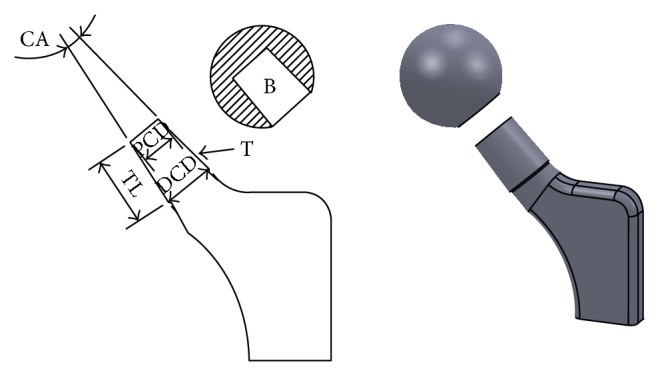
Diagrammatic representation of a femoral head-neck taper junction consisting of a female bore (B) and male trunnion (T). The trunnion length (TL), proximal cone diameter (PCD), distal cone diameter (DCD), and cone angle (CA) are displayed.

The Oxford English Dictionary defines the word “taper” as “reduce in thickness towards one end” [22]. A more descriptive definition is the uniform change in diameter of a cylindrical object measured along its axis. The concept of a Morse taper [23] is that of a cone within a cone—the trunnion (male portion) and the bore (female portion)—and the stresses created by the compression of the wall of the bore by the trunnion causes an interference fit (Figure 1) and cold-welding between the two components, which increases during physiologic loading [2]. The radial compressive stress causing friction between the taper surfaces also provides resistance to separation at the head-neck junction. The original Morse taper cone angle described was 2°50′ [23] but within Orthopaedics, the term Morse taper is loosely used to encompass tapers of all angles that result in cold-welding of one element upon another.

## 3. Pathogenesis

Metal alloys traditionally used to manufacture THA implants offer good biocompatibility, corrosion resistance, and mechanical properties [24]. The head-neck junctions of femoral stems are usually composed of titanium alloy (Ti-6Al-4V) or cobalt-chromium-molybdenum alloy (commonly known as cobalt-chrome, CoCr), both of which form a protective surface oxide layer through a process of self-passivation. This aids in resisting corrosion. Cyclic loads through the hip joint may induce small oscillatory motions between the head and neck at the taper. This results in disruption of the passive oxide layer by fretting [1, 5, 6]. When the underlying metal is exposed, repassivation occurs, altering the passivation potential and acidity of the local fluid environment. Eventually, the ability to form a protective surface oxide layer is depleted, and this makes the exposed metal more susceptible to corrosion [2, 25–27].

Taper corrosion includes a complex interaction of crevice corrosion, initiated by changes in local chemistry within crevices, and fretting, which disrupts the protective oxide layer on the taper [4, 28, 29]. This process has also been termed “mechanically assisted crevice corrosion” (MACC) [30]. In addition, the presence in the body of mixed metal systems, such as Ti-6Al-4V stems with CoCr heads, can induce galvanic corrosion. Corrosion appears to cause more loss of material from the bore of the head than from the trunnion [1, 4–6].

MACC can potentially produce by-products at the head-neck junction, primarily metal ions, which can migrate locally and systemically. This may cause elevated serum metal ion levels in extensively corroded tapers and is thought to initiate a cascade of events leading to ALTRs [1, 2]. These include macrophage responses to particulate corrosion products, which are more likely to be generated by fretting wear, and a lymphocyte-dominated response to metal ion complexes, which in some cases may lead to necrosis of periprosthetic tissues, local synovitis, osteolysis, component loosening, and early failure [2, 31].

The histopathology is characterised by chronic inflammation and necrosis of periprosthetic soft tissues [1]. Lymphocytes, plasmocytes, and eosinophils may be found in viable areas of pseudocapsule and adjacent muscle [31]. Both titanium oxide particles [31] and CoCr corrosion products [32, 33] elicit a local macrophage-mediated inflammatory response, associated with increased release of inflammatory cytokines interleukin-6 (IL-6) and tumour necrosis factor-alpha (TNF-*α*) [34, 35]. This may lead to osteolysis and contribute to loosening [32].

## 4. Etiology

### 4.1. Taper Design

**Figure 2:**
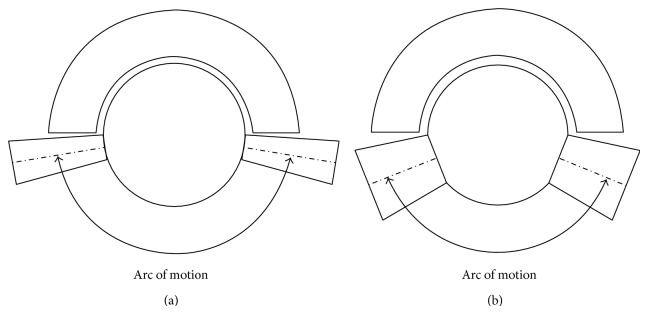
The effect of a long, thin taper junction (a) and a short, thick taper junction (b) on impingement and range of motion.

Tapers exist in multiple sizes and angles. Studies have shown that larger diameter and longer tapers with circular necks result in a reduction of the range of motion, compared to a smaller diameter and shorter taper with a trapezoidal neck [36–38]. With the former, there is reduced arc of motion before impingement occurs and hence increased risk of dislocation (Figure 2).

**Figure 3:**
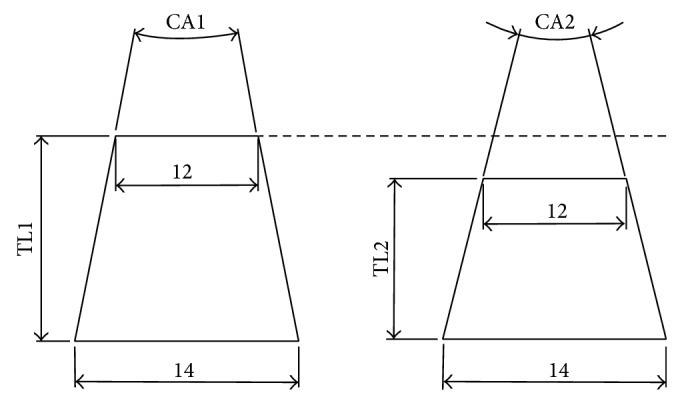
Illustration to demonstrate the effect that trunnion length has on cone angle with a constant proximal and distal diameter, for example, in a “12/14” taper. With increasing trunnion length (TL1 > TL2), the cone angle decreases (CA1 < CA2) assuming the proximal cone diameter and distal cone diameter remain constant.

Trunnions are now manufactured shorter in order to reduce the dislocation risk; with time, 14 mm/16 mm trunnions have been replaced by narrower 12 mm/14 mm trunnions [18]. An important concept to understand is that not all “12/14” tapers have the same cone angle, as the angle is also dependent on the trunnion length (Figure 3).

It is not entirely clear what taper geometric parameters influence its corrosion. This is evident by the numerous varieties available from the different implant manufacturers. Shorter trunnions which tend to sit entirely within the bore are thought to increase edge loading at its base. This may increase local stresses and cause increased damage to the taper [18]. Narrower trunnions provide less surface area for interference fit and may increase micromotion at the head-neck junction. Whilst protecting against dislocations [39, 40], maximizing the head-neck ratio with the use of shorter, smaller tapers may also result in increased fretting and corrosion [2]. In an in vitro study analysing tapers with different surface finishes and contact areas, surface roughness on the head taper was significantly increased after 10^7^ loading cycles, when the taper contact area was reduced and when necks with roughened trunnions were used [41]. A recent retrieval study [19] of 40 femoral heads has suggested the contrary—shorter, thinner tapers had lower fretting scores compared to longer, thicker tapers, based on a previously described scoring system [4] and on scans of negative molds of the female tapers. The findings in that study [19] suggested that the larger the available surface area, the greater the area over which fretting can occur.

**Figure 4:**
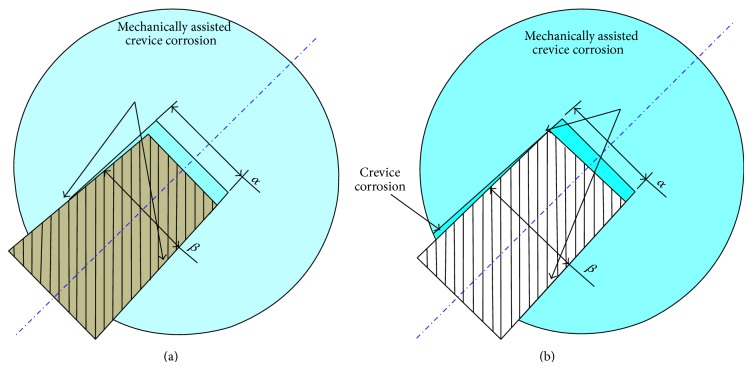
Mechanically assisted crevice corrosion in different scenarios of angular mismatch: (a) head angle, *α*, greater than neck angle, *β*; and (b) head angle, *α*, less than neck angle, *β*; crevice corrosion can potentially occur in this design due to the corrosive fluid present at the interface.

The taper cone angle varies according to the design; in one study, the taper angle was measured as 4.0°, 6.0°, and 5.6° in types 1, 11/13, and 12/14 tapers, respectively [19]. There were no associations established between taper angles and corrosion scores. Another study looked at the effects of angular mismatch at the taper junction [42], by simulating a taper junction on a computer model. Micromotion at the taper was independent of angular mismatches up to 0.075°, but increase in micromotion was observed above this threshold. The current industry accepted tolerance is 0.0167° [42], which is lower than the threshold in the quoted study. This area still needs further investigation as there are theoretically increased risks of corrosion with angular mismatch (Figures 4(a) and 4(b)).

Most commercially available trunnions now have surface ridges that can deform during impaction of the ceramic head, providing a close fit to the shape of the bore. Some authors have postulated that this leads to altered contact stresses and thus increased trunnion wear [18, 41]. In one study looking at 161 failed MoM THAs, the authors suggested that these ridges allow fluid to travel along the microthread of the trunnion and are “detrimental” [30]. Further investigation is required to determine whether different taper designs are required for metal and ceramic heads.

### 4.2. Material

#### 4.2.1. Head

Ceramic is known for its inert and electrically insulating properties. In an in vitro study analysing fretting corrosion between zirconia ceramic heads and cobalt-alloy stems compared to metal (cobalt-alloy) heads and cobalt-alloy stems, there was less fretting corrosion in the ceramic group [43]. Limitations of this study included its single stem/head design nature and the fact that the use of zirconia heads have generally been discontinued. A more recent retrieval study [20] analysed fretting corrosion in matched groups of 50 alumina ceramic heads and 50 CoCr heads on metal tapers. The corrosion scores were lower for the stems in the ceramic group, suggesting that taper corrosion may be mitigated but not eliminated by using ceramic heads. As the quality of ceramic has improved, the risk of ceramic fractures has decreased to between 0.02 and 0.1% [44]. However, concerns exist as to whether ceramic heads may safely be reimplanted onto femoral stems that are not being revised. The concern is that the trunnion is likely to be damaged during removal of the femoral head and damaged areas may lead to stress risers responsible for the initiation of a fracture in a newly implanted ceramic head [45, 46]. If the head is not securely fixed onto the damaged taper, there may be a risk of increased wear due to the abnormal movements at the head-neck junction. In a study reporting 61 cases in which alumina heads were reimplanted onto well-fixed titanium stems at revision of ceramic-on-ceramic prostheses, no fractures of the head had occurred at a mean of 88-month follow-up [47]. Those authors suggested that this approach is acceptable so long as taper inspection revealed only minor scratches and the newly implanted ceramic head had a stable fit on the taper. As this practice is against the manufacturers' recommendations, the surgeon should consider the potential legal implications in cases of subsequent head fracture.

### 4.3. Head-Neck Couple

Micromotion at the head-neck junction may induce fretting corrosion and contribute to the total failure of the implant [48]. In a study where a Ti-6Al-4V femoral neck and a 316L stainless steel head were examined under axial fretting in ambient air and an artificial physiologic medium [48], 316L steel showed fewer emitted wear particles under fretting corrosion conditions than the titanium alloy. In another study looking at how fretting corrosion is affected by different material coupling combinations (Ti-6Al-4V/Ti-6Al-4V, CoCr/Ti-6Al-4V, and CoCr/CoCr) [49], the Ti-6Al-4V/Ti-6Al-4V couple showed the most significant surface damage under axial loading. However, such in vitro studies are limited as they cannot precisely duplicate all the in vivo conditions.

In another retrieval study, titanium-titanium interfaces demonstrated the least amount of fretting when compared to CoCr/CoCr and CoCr/titanium interfaces [19]. The authors suggest that this may reflect the ability of titanium-titanium interfaces to form a better interference fit via cold-welding. A better interference fit, however, may be a problem during revision surgery, as multiple attempts at dislodging the femoral head from the stem may end up damaging the trunnion, or the head may not disengage from the stem, necessitating revision of a (well-fixed) stem [50]. Titanium alloy is not a commonly used material for femoral head manufacturing due to its lower modulus of elasticity and inferior wear properties compared to Co-Cr or ceramic, so this may not be so clinically relevant [51, 52].

In another retrieval study examining 269 implants, mild to moderate trunnion fretting and corrosion damage was observed in 38% (45/118) of CoCr head/titanium-alloy stem couple and 21% (32/151) of CoCr head/CoCr stem couple [53]. In a large multicentre retrieval study, moderate to severe corrosion was noted in 28% (65/231) of heads of similar alloy couples compared to 42% (97/231) of heads of mixed alloy couples [4]. In both studies the differences were statistically significant but no mention was made of what type of articulating surfaces was used. Dissimilar alloy pairing between the head and stem causes more damage at both the head and taper when compared to components with similar alloy pairing [4] as the former is more susceptible to galvanic corrosion. Interestingly, several multicentre retrieval studies have reported higher rates and more severe corrosion and fretting in the head taper compared to the neck [4, 54]. In addition, different grades of metallic alloys with different chemical compositions such as Ti-6Al-4V and Ti-6Al-4V (ELI grade) possess different mechanical and surface properties and therefore may have different levels of corrosion resistance.

### 4.4. Femoral Head Size

Larger heads have increased jump distance, larger femoral head-neck ratio, and a greater impingement-free range of motion [16, 39, 55]. The Medicare database showed that the use of larger head sizes (32 mm or greater) over the course of a decade resulted in a decrease in dislocation rate of about 50% [56]. However, the recent trend of using larger femoral heads to reduce risks of dislocation may result in higher stresses at the femoral head-neck junction [18]. In a retrieval study looking at CoCr alloy head and stem pairings featuring a 12/14 mm taper in metal-on-polyethylene articulations, there was a significant increase in taper corrosion scores of 36 mm heads compared with 28 mm heads [3]. In another retrieval study looking at 90 modular implants, corrosion damage of the trunnion varied with type of articulation and head size [57]. In a retrieval analysis of 100 taper junctions from metal-on-polyethylene articulations, 16% of the tapers had severe or moderate corrosion [4] independent of femoral head size [54]. Taper corrosion was most strongly correlated to the severity of the abrasive wear affecting the surface of the CoCr head. Femoral head size only became an important cofactor in predicting corrosion in the presence of abrasive wear of the surface of the head. The authors postulated that the abrasive wear seen is secondary to third-body damage, which is common in many large diameter polyethylene liners and this would lead to increases in frictional torques.

The possible influence of head size on taper corrosion can be speculated from the reported survival of MoM THAs in the Australian Joint Replacement Registry. An increased rate of revision was associated with the use of head sizes >32 mm in MoM THAs whereas the cumulative revision rates of those with head size <32 mm were comparable to their metal-on-polyethylene counterparts [15]. The possible role of the taper junction is also suggested by the higher revision rates of large MoM THAs compared to their hip resurfacing counterparts in the registry report. For failed MoM THA, material loss occurs preponderantly from wear of the metal bearing surface and retrieval analysis showed that the volumetric loss at the taper junction represented only 6.1% of the material loss in these cases [58].

### 4.5. Others

Other factors that may affect taper corrosion include patient body mass index (BMI), lateral offset, varus femoral stem positioning, longer necks, time since implantation, and inconsistency in assembly of modular heads including the force of impaction, the vector of the applied force, and contamination of the interface [4, 8, 21, 54]. It is interesting to note that the observation of trunnion-related revisions is relatively recent and has coincided with the more widespread use of smaller incisions and operative approaches that may make correct trunnion assembly more difficult.

## 5. Clinical

The true incidence of clinically relevant taper corrosion is not known, but in one study, 10 of 569 (1.8%) revision THAs performed were due to complications arising from corrosion at the head-neck taper in metal-on-polyethylene articulations [1]. Diagnosis is often difficult unless significant associated ALTRs occur. A painful THA should have taper corrosion on its list of differential diagnoses even though more common causes such as aseptic loosening secondary to particulate debris from the bearing surface and infection should be higher on the list.

Taper corrosion may be suggested by elevated serum cobalt and chromium levels, particularly a differential elevation of serum cobalt levels with respect to chromium levels [1, 17]. As the clinical picture may be unclear, elevation of the erythrocyte sedimentation rate (ESR) and C-reactive protein (CRP) may be a guide to hip aspiration to exclude infection, which provides the opportunity for joint fluid metal ion analysis. Ultrasound is an effective screening tool, and metal-artifact reduction magnetic resonance imaging (MRI) provides a visual assessment of the extent, if any, of synovitis, osteolysis, muscle/tendon destruction, periprosthetic fluid collections, and pseudotumour formation [59].

## 6. Treatment

**Figure 5:**
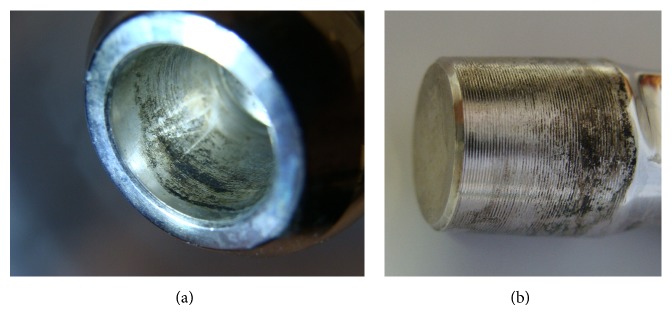
Fretting and corrosion damage on the (a) bore of a CoCr femoral head and (b) Ti-alloy trunnion from a retrieved failed metal-on-polyethylene THR.

The surgeon has to weigh the potential benefits of revising a well-fixed stem which has trunnion damage against the potential morbidity associated with stem revision. Avoiding stem revision reduces surgical time, blood loss, and complications. To avoid the difficult revision of a well-fixed stem, the common practice is to place a new head on a retained stem. This should only be performed when trunnion corrosion is macroscopically mild (visible but not palpable defects) and when an interference fit can be achieved with the new head (Figures 5(a) and 5(b)). Although the long-term consequence of this practice is unknown, an in vitro and retrieval study favours this [60]. In a study in which retrieved femoral heads assembled onto unimplanted Ti-6Al-4V trunnions were mechanically tested, the authors concluded that the torsional properties of the taper junctions were not significantly influenced by mild or moderate grades of corrosion [60]. If the stem is to be retained, we advocate intraoperative trunnion protection with the use of a cut-out suction tubing or plastic syringe [61]. However, given the lack of consensus on the issue of how much taper damage warrants stem revision, and how much taper damage is acceptable, the decision to retain or revise a stable stem which has taper damage should be left to the judgment of the treating surgeon.

The use of ceramic heads may reduce future problems with taper corrosion but concerns exist in the revision scenario of reimplantation of ceramic heads onto damaged tapers [1, 20, 62, 63]. It has been suggested that the use of a titanium alloy adaptor sleeve might help prevent the recurrence of ALTRs by way of changing the material combination at the head-neck junction to a Ti-6Al-4V/Ti-6Al-4V or Ti-6Al-4V/CoCr pairing depending on the material of the stem [1, 19]. This has substantial added costs and may not be a viable option in every hospital.

It is imperative to ensure that any femoral head implanted onto a trunnion should be compatible with the design of the trunnion, as mismatch may contribute to taper corrosion from increased micromotion [42]. It has been noted that 12/14 trunnions from different implants and companies differ subtly in dimensions and cone angles [23]. Only one of the orthopaedic companies contacted by the authors requesting details of trunnion dimensions agreed to have the data made available for clinicians through a forum like this journal. To illustrate the importance of such information we compared the 12/14 taper of company A (trunnion length, 11.80 mm; proximal cone diameter, 12.60 mm; distal cone diameter, 13.70 mm) and company B (trunnion length, 11.20 mm; proximal cone diameter, 12.60 mm; distal cone diameter, 13.65 mm). The cone angles of these two implants are 5°42′30′′ and 5°67′, equaling a difference of 0.412° or more than five times the 0.075° threshold mismatch above which micromotion is increased at the head-neck junction [42]. Therefore, tapers should never be mismatched, even if they are from the same manufacturer, or if the taper design (e.g., 12/14) quoted is the same. The process of applying a femoral head onto a trunnion needs attention; we advocate the cleaning and drying of the surfaces prior to application to avoid any fluid or tissue that may lodge between the two surfaces, preventing adequate interlocking of the taper [64].

## 7. Summary

Although modular hips were introduced in the 80s and taper corrosion was reported in the early 90s, the contribution of fretting and corrosion at the taper junction [51, 65] to implant failure is now being increasingly recognised. The severity of early corrosion-related failure of modular femoral stem-neck junction is a potential reason for concern [66], and the surgeon should be aware of the potential drawbacks of modularity in THA. Factors under the surgeon's influence include choice of femoral head material, femoral head size, engagement of the taper in an environment free from blood or fat, force and technique of impaction [64, 67, 68], and avoiding angular mismatch at the taper junction [69]. The surgeon's choice of stem will govern the taper design, but design is under the control of the manufacturers.

Data on taper corrosion are generated from in vitro and retrieval studies. Given that in vitro studies cannot precisely reproduce all the in vivo conditions, these results should be interpreted with caution. Retrieval studies also have limitations as they involve analysis of clinically failed implants and these findings may not necessarily reflect the well-functioning, unrevised implants [20]. In addition, among the implants in a given study, there is often heterogeneity in the bearing surfaces used, head size, offset, stem type, stem alloy, and implant manufacturer which may have implications on taper corrosion.

There is likely to be a wide spectrum when it comes to taper corrosion, ranging from mild corrosion with no tissue reaction and little or no clinical implications to substantial ALTRs with abductor deficiency, metallosis, and pseudotumour formation with significant clinical implications akin to that seen in hips with MoM bearing surfaces.

Although taper corrosion is a real entity, one must beware of overdiagnosing this condition based on the recent increasing interest. Although it was not well appreciated in the past, it is unlikely to pose a major clinical issue at present, and potential unnecessary changes in practice should be avoided until there is clear evidence to suggest the contrary.

## References

[1] Cooper H. J., Della Valle C. J., Berger R. A. (2012). Corrosion at the head-neck taper as a cause for adverse local tissue reactions after total hip arthroplasty. *Journal of Bone and Joint Surgery—American Volume*.

[2] Srinivasan A., Jung E., Levine B. R. (2012). Modularity of the femoral component in total hip arthroplasty. *Journal of the American Academy of Orthopaedic Surgeons*.

[3] Dyrkacz R. M. R., Brandt J.-M., Ojo O. A., Turgeon T. R., Wyss U. P. (2013). The influence of head size on corrosion and fretting behaviour at the head-neck interface of artificial hip joints. *Journal of Arthroplasty*.

[4] Goldberg J. R., Gilbert J. L., Jacobs J. J., Bauer T. W., Paprosky W., Leurgans S. (2002). A multicenter retrieval study of the taper interfaces of modular hip prostheses. *Clinical Orthopaedics and Related Research*.

[5] Higgs G. B., Hanzlik J. A., MacDonald D. W. (2013). Is increased modularity associated with increased fretting and corrosion damage in Metal-on-Metal Total Hip Arthroplasty devices? A retrieval study. *Journal of Arthroplasty*.

[6] Higgs G. B., Hanzlik J. A., MacDonald D. W., Kurtz S. M., Greenwald A. S., Mihalko W. M., Lemons J. (2013). Method of characterizing fretting and corrosion at the various taper connections of retrieved modular components from metal-on-metal total hip arthroplasty. *Metal-on-Metal Total Hip Replacement Devices*.

[7] Collier J. P., Surprenant V. A., Jensen R. E., Mayor M. B. (1991). Corrosion at the interface of cobalt-alloy heads on titanium-alloy stems. *Clinical Orthopaedics and Related Research*.

[8] Gilbert J. L., Buckley C. A., Jacobs J. J. (1993). In vivo corrosion of modular hip prosthesis components in mixed and similar metal combinations. The effect of crevice, stress, motion, and alloy coupling. *Journal of Biomedical Materials Research*.

[9] Kawalec J. S., Brown S. A., Payer J. H., Merritt K. (1995). Mixed-metal fretting corrosion of Ti6Al4V and wrought cobalt alloy. *Journal of Biomedical Materials Research*.

[10] D'Lima D. D., Hermida J. C., Chen P. C., Colwell C. W. (2003). Polyethylene cross-linking by two different methods reduces acetabular liner wear in a hip joint wear simulator. *Journal of Orthopaedic Research*.

[11] Thomas G. E. R., Simpson D. J., Mehmood S. (2011). The seven-year wear of highly cross-linked polyethylene in total hip arthroplasty: a double-blind, randomized controlled trial using radiostereometric analysis. *Journal of Bone and Joint Surgery—American Volume*.

[12] Mutimer J., Devane P. A., Adams K., Horne J. G. (2010). Highly crosslinked polyethylene reduces wear in total hip arthroplasty at 5 years. *Clinical Orthopaedics and Related Research*.

[13] Hannouche D., Zaoui A., Zadegan F., Sedel L., Nizard R. (2011). Thirty years of experience with alumina-on-alumina bearings in total hip arthroplasty. *International Orthopaedics*.

[14] Australian Orthopaedic Association National Joint Replacement Registry (2013). Hip and knee arthroplasty. *Annual Report*.

[15] Australian Orthopaedic Association National Joint Replacement Registry (2012). *Hip and Knee Arthroplasty. Annual Report 2012*.

[16] Howie D. W., Holubowycz O. T., Middleton R. (2012). Large femoral heads decrease the incidence of dislocation after total hip arthroplasty: a randomized controlled trial. *The Journal of Bone and Joint Surgery—American Volume*.

[17] Garbuz D. S., Tanzer M., Greidanus N. V., Masri B. A., Duncan C. P. (2010). The john charnley award: metal-on-metal hip resurfacing versus large-diameter head metal-on-metal total hip arthroplasty: a randomized clinical trial. *Clinical Orthopaedics and Related Research*.

[18] Langton D. J., Sidaginamale R., Lord J. K., Nargol A. V., Joyce T. J. (2012). Taper junction failure in large-diametermetal-on-metal bearings. *Bone and Joint Research*.

[19] Nassif N. A., Nawabi D. H., Stoner K., Elpers M., Wright T., Padgett D. E. (2014). Taper design affects failure of large-head metal-on-metal total hip replacements. *Clinical Orthopaedics and Related Research*.

[20] Kurtz S. M., Kocagöz S. B., Hanzlik J. A. (2013). Do ceramic femoral heads reduce taper fretting corrosion in hip arthroplasty? A retrieval study. *Clinical Orthopaedics and Related Research*.

[21] Brown S. A., Flemming C. A. C., Kawalec J. S. (1995). Fretting corrosion accelerates crevice corrosion of modular hip tapers. *Journal of Applied Biomaterials*.

[22] Oxford Dictionaries http://www.oxforddictionaries.com.

[23] Hernigou P., Queinnec S., Flouzat Lachaniette C. H. (2013). One hundred and fifty years of history of the Morse taper: from Stephen A. Morse in 1864 to complications related to modularity in hip arthroplasty. *International Orthopaedics*.

[24] Hoeppner D. W., Chandrasekaran V. (1994). Fretting in orthopaedic implants: a review. *Wear*.

[25] Sivakumar B., Kumar S., Sankara Narayanan T. S. N. (2011). Fretting corrosion behaviour of Ti–6Al–4V alloy in artificial saliva containing varying concentrations of fluoride ions. *Wear*.

[26] Rodrigues D. C., Urban R. M., Jacobs J. J., Gilbert J. L. (2009). In vivo severe corrosion and hydrogen embrittlement of retrieved modular body titanium alloy hip-implants. *Journal of Biomedical Materials Research Part B: Applied Biomaterials*.

[27] Virtanen S., Milošev I., Gomez-Barrena E., Trebše R., Salo J., Konttinen Y. T. (2008). Special modes of corrosion under physiological and simulated physiological conditions. *Acta Biomaterialia*.

[28] Jacobs J. J., Gilbert J. L., Urban R. M. (1998). Corrosion of metal orthopaedic implants. *The Journal of Bone & Joint Surgery—American Volume*.

[29] Gilbert J. L., Jacobs J. J., Marlowe D., Parr J., Mayor M. B. (1997). The mechanical and electrochemical processes associated with taper fretting crevice corrosion: a review. *Modularity of Orthopedic Implants, ASTM STP 1301*.

[30] Hexter A., Panagiotidou A., Singh J., Skinner J., Hart A. (2013). Mechanism of corrosion in large diameter head metal-on-metal total hip arthroplasty: a retrieval analysis of 161 components. *The Bone & Joint Journal—British Volume*.

[31] Urban R. M., Hall D. J., Cooper H. J., Valle C. J. D., Galante J. O., Jacobs J. J. Local histological and systemic effects of metal corrosion products.

[32] Lee S.-H., Brennan F. R., Jacobs J. J., Urban R. M., Ragasa D. R., Glant T. T. (1997). Human monocyte/macrophage response to cobalt-chromium corrosion products and titanium particles in patients with total joint replacements. *Journal of Orthopaedic Research*.

[33] Howie D. W., Vernon-Roberts B. (1988). The synovial response to intraarticular cobalt-chrome wear particles. *Clinical Orthopaedics and Related Research*.

[34] Trindade M. C. D., Lind M., Sun D., Schurman D. J., Goodman S. B., Smith R. L. (2001). In vitro reaction to orthopaedic biomaterials by macrophages and lymphocytes isolated from patients undergoing revision surgery. *Biomaterials*.

[35] Rogers S. D., Howie D. W., Graves S. E., Pearcy M. J., Haynes D. R. (1997). In vitro human monocyte response to wear particles of titanium alloy containing vanadium or niobium. *Journal of Bone and Joint Surgery—British Volume*.

[36] Barrack R. L., Butler R. A., Laster D. R., Andrews P. (2001). Stem design and dislocation after revision total hip arthroplasty: clinical results and computer modeling. *Journal of Arthroplasty*.

[37] Padgett D. E., Lipman J., Robie B., Nestor B. J. (2006). Influence of total hip design on dislocation: a computer model and clinical analysis. *Clinical Orthopaedics and Related Research*.

[38] Malik A., Maheshwari A., Dorr L. D. (2007). Impingement with total hip replacement. *The Journal of Bone & Joint Surgery—American Volume*.

[39] Rodriguez J. A., Rathod P. A. (2012). Large diameter heads: is bigger always better?. *The Journal of Bone and Joint Surgery—British Volume*.

[40] Berry D. J., von Knoch M., Schleck C. D., Harmsen W. S. (2005). Effect of femoral head diameter and operative approach on risk of dislocation after primary total hip arthroplasty. *The Journal of Bone & Joint Surgery—American Volume*.

[41] Panagiotidou A., Meswania J., Hua J., Muirhead-Allwood S., Hart A., Blunn G. (2013). Enhanced wear and corrosion in modular tapers in total hip replacement is associated with the contact area and surface topography. *Journal of Orthopaedic Research*.

[42] Parekh J., Jones H., Chan N., Noble P. (2013). Effect of angular mismatch tolerance on trunnion micro-motion in metal-on-metal THA designs. *The Bone & Joint Journal—British Volume*.

[43] Hallab N. J., Messina C., Skipor A., Jacobs J. J. (2004). Differences in the fretting corrosion of metal-metal and ceramic-metal modular junctions of total hip replacements. *Journal of Orthopaedic Research*.

[44] Hannouche D., Nich C., Bizot P., Meunier A., Nizard R., Sedel L. (2003). Fractures of ceramic bearings: history and present status. *Clinical Orthopaedics and Related Research*.

[45] Willmann G. (1998). Ceramics for total hip replacement—what a surgeon should know. *Orthopedics*.

[46] Willmann G. (2000). Cerami femoral head retrieval data. *Clinical Orthopaedics and Related Research*.

[47] Hannouche D., Delambre J., Zadegan F., Sedel L., Nizard R. (2010). Is there a risk in placing a ceramic head on a previously implanted trunion?. *Clinical Orthopaedics and Related Research*.

[48] Duisabeau L., Combrade P., Forest B. (2004). Environmental effect on fretting of metallic materials for orthopaedic implants. *Wear*.

[49] Swaminathan V., Gilbert J. L. (2012). Fretting corrosion of CoCrMo and Ti6Al4V interfaces. *Biomaterials*.

[50] Kop A. M., Keogh C., Swarts E. (2012). Proximal component modularity in THA—at what cost? An implant retrieval study. *Clinical Orthopaedics and Related Research*.

[51] Manivasagam G., Dhinasekaran D., Rajamanickam A. (2010). Biomedical implants: corrosion and its prevention—a review. *Recent Patents on Corrosion Science*.

[52] Brummitt I. K., Hardaker C. S., McCullagh P. J. J., Drabu K. J., Smith R. A. (1996). Effect of counterface material on the characteristics of retrieved uncemented cobalt-chromium and titanium alloy total hip replacements. *Proceedings of the Institution of Mechanical Engineers, Part H*.

[53] Urban R. M., Hall D. J., Gilbert J. L. Are fretting and corrosion reduced in contemporary head/neck modular junctions?.

[54] Moga I., Harington M. A., Noble P. C. Variables influencing tribo-corrosion of modular junctions in metal-on-polyethylene THR?.

[55] Garbuz D. S., Masri B. A., Duncan C. P. (2012). The Frank Stinchfield award: dislocation in revision THA: do large heads (36 and 40 mm) result in reduced dislocation rates in a randomized clinical trial?. *Clinical Orthopaedics and Related Research*.

[56] Malkani A. L., Ong K. L., Lau E., Kurtz S. M., Justice B. J., Manley M. T. (2010). Early- and late-term dislocation risk after primary hip arthroplasty in the medicare population. *Journal of Arthroplasty*.

[57] Moga I., Harrington M. A., Ismaily S., Noble P. (2013). Trunnion surface damage in THR: MPE *vs* MOM articulations. *The Bone & Joint Journal—British Volume*.

[58] Matthies A., Bills P. J., Racasan R. Analysis of the taper supports retention of a well-fixed stem in revision surgery of metal-on-metal hip replacements.

[59] Toms A. P., Marshall T. J., Cahir J. (2008). MRI of early symptomatic metal-on-metal total hip arthroplasty: a retrospective review of radiological findings in 20 hips. *Clinical Radiology*.

[60] Alexander J., Hexter A., Ismaily S., Hart A., Noble P. (2013). The effect of tribo-chemical damage on mechanical performance of TIAlV-CoCr taper junctions. *Poster*.

[61] Puolakka T. J. S., Halonen P. J., Oksa J., Pajamäki K. J. J. (2002). Protection of femoral neck taper in revision of acetabular component. *Journal of Arthroplasty*.

[62] Meneghini R. M., Hallab N. J., Jacobs J. J. (2012). Evaluation and treatment of painful total hip arthroplasties with modular metal taper junctions. *Orthopedics*.

[63] Traina F., Tassinari E., De Fine M., Bordini B., Toni A. (2011). Revision of ceramic hip replacements for fracture of a ceramic component: AAOS exhibit selection. *The Journal of Bone and Joint Surgery—American Volume*.

[64] Mroczkowski M. L., Hertzler J. S., Humphrey S. M., Johnson T., Blanchard C. R. (2006). Effect of impact assembly on the fretting corrosion of modular hip tapers. *Journal of Orthopaedic Research*.

[65] Engh C. A., Ho H., Padgett D. E. (2014). The surgical options and clinical evidence for treatment of wear or corrosion occurring with THA or TKA. *Clinical Orthopaedics and Related Research®*.

[66] Meftah M., Haleem A. M., Burn M. B., Smith K. M., Incavo S. J. (2014). Early corrosion-related failure of the Rejuvenate modular total hip replacement. *Journal of Bone and Joint Surgery—American Volume*.

[67] Vail T. P. Complications: metal-on-metal articulations—design and technique related factors.

[68] Rehmer A., Bishop N. E., Morlock M. M. (2012). Influence of assembly procedure and material combination on the strength of the taper connection at the head-neck junction of modular hip endoprostheses. *Clinical Biomechanics*.

[69] Miloŝev L., Antoliĉ V., Minoviĉ A. (2000). Extensive metallosis and necrosis in failed prostheses with cemented titanium-alloy stems and ceramic heads. *The Journal of Bone & Joint Surgery—British Volume*.

